# SLAMF1-derived peptide exhibits cardio protection after permanent left anterior descending artery ligation in mice

**DOI:** 10.3389/fimmu.2024.1383505

**Published:** 2024-04-15

**Authors:** Maria Belland Olsen, Xiang Yi Kong, Mieke C. Louwe, Knut H. Lauritzen, Ylva Schanke, Ole Jørgen Kaasbøll, Håvard Attramadal, Jonas Øgaard, Sverre Holm, Pål Aukrust, Liv Ryan, Terje Espevik, Maria Yurchenko, Bente Halvorsen

**Affiliations:** ^1^ Research Institute of Internal Medicine, Oslo University Hospital, Rikshospitalet, Oslo, Norway; ^2^ Faculty of Medicine, Institute of Clinical Medicine, University of Oslo, Oslo, Norway; ^3^ Institute for Surgical Research, Oslo University Hospital, Rikshospitalet, Oslo, Norway; ^4^ Centre of Molecular Inflammation Research, Department of Clinical and Molecular Medicine, Norwegian University of Science and Technology, Trondheim, Norway; ^5^ Department of Infectious Diseases, Clinic of Medicine, St. Olav’s Hospital HF, Trondheim University Hospital, Trondheim, Norway

**Keywords:** myocardial infarction, inflammation, TLR4, SLAMF1, P7

## Abstract

Acute myocardial infarction (MI) results in tissue damage to affected areas of the myocardium. The initial inflammatory response is the most damaging for residual cardiac function, while at later stages inflammation is a prerequisite for proper healing and scar formation. Balancing the extent and duration of inflammation during various stages after MI is thus pivotal for preserving cardiac function. Recently, a signaling lymphocytic activation molecule 1 (SLAMF1)-derived peptide (P7) was shown to reduce the secretion of inflammatory cytokines and protected against acute lipopolysaccharide-induced death in mice. In the present study, we experimentally induced MI by permanent ligation of the left anterior descending artery (LAD) in mice and explored the beneficial effect of immediately administering P7, with the aim of dampening the initial inflammatory phase without compromising the healing and remodeling phase. Blood samples taken 9 h post-LAD surgery and P7 administration dampened the secretion of inflammatory cytokines, but this dampening effect of P7 was diminished after 3 days. Echocardiography revealed less deterioration of cardiac contraction in mice receiving P7. In line with this, less myocardial damage was observed histologically in P7-treated mice. In conclusion, the administration of a SLAMF1-derived peptide (P7) immediately after induction of MI reduces the initial myocardial inflammation, reduces infarct expansion, and leads to less deterioration of cardiac contraction.

## Introduction

Acute myocardial infarction (MI) occurs when there is a sudden, insufficient supply of blood to specific areas of the myocardium, leading to tissue damage. Inflammation plays an important role in facilitating proper healing and scar formation following MI but is also crucial in promoting tissue damage in areas at risk as well as mediating maladaptive myocardial remodeling predisposing to myocardial failure (1). Thus, balancing the extent and duration of inflammation during various stages of tissue damage, healing, and remodeling is pivotal for preserving cardiac function after MI. During the immediate inflammatory phase following MI, where ischemia–reperfusion (I/R) injury is of major importance, there is a significant influx of immune cells, which appears to be the most detrimental phase for cardiac function (1). In experimental murine models of MI, the neutralization of early-phase inflammatory cytokines such as interleukin (IL)-1α, IL-1β, and IL-18 preserved left ventricular (LV) function, suggesting a beneficial effect of reducing inflammation during this phase (2–4). We have recently shown a similar pattern in humans by blocking IL-6/IL-6 receptor interaction in MI patients (5).

Blocking the upstream inflammatory cascade to prevent the release of inflammatory cytokines could be an attractive approach for myocardial protection following MI. We have recently developed a signaling lymphocytic activation molecule 1 (SLAMF1)-derived peptide (P7) that prevents Toll-like receptor (TLR)4-mediated signaling involving the modulation of the interaction between SLAMF1 and the TLR4 adaptor protein TRIF-related adapter molecule (TRAM). The P7 peptide also interferes with TIRAP–MyD88 interactions (6). In mice subjected to lipopolysaccharide (LPS)-induced shock, P7 decreased the secretion of inflammatory cytokines and prevented animal death (6).

TLR4-signaling, induced by danger-associated molecular patterns (DAMPs), e.g., heat shock proteins, high mobility group box-1 and adenosine triphosphate, released by cell damage and cell death minutes to hours and even days within the myocardium following an ischemic event, is activated during MI (7). Thus, downstream signaling of TLR4 increases the release of inflammatory cytokines, causing additional damage to the already injured myocardium (8). We hypothesized that administration of P7 immediately after MI would improve the long-term residual cardiac function by dampening the initial inflammatory phase. Due to the expected short lifespan of P7 (6), this experimental setup would not compromise inflammation during the healing and remodeling phase.

## Materials and methods

### SLAMF1-derived peptide

The synthetic peptide was from GenSript (Netherlands), comprised of a SLAMF1-derived sequence (10 amino acids), glycin as a linker, and penetratin as a cell-penetrating peptide to ensure intracellular delivery. The P7 (P7-Pen) sequence is ITVYASVTLT G RQIKIWFQNRRMKWKK. The peptide that has got N-terminal acetylation and C-terminal amidation during synthesis, was provided at >90% purity, after guaranteed TFA removal, and controlled for the absence of endotoxin (less than <10 EU/mg). The peptide was stored as in powder form at -20°C and diluted in sterile-filtered distilled water immediately before use. The peptide was shown to be non-toxic for the mice at 8.5-mg/kg or 17-mg/mL concentrations (6).

### Animals

This study has been approved by the Norwegian National Animal Research Authority with project license number FOTS 28338. All animal experiments were performed in accordance with the European Directive 2010/63/EU and conducted in accordance with Animals in Research: Reporting *In Vivo* Experiments (ARRIVE) guidelines. For all parts of this study, the investigators were blinded in relation to the treatment group.

Experimental MI was induced by permanent coronary artery occlusion without ventilation as described in detail elsewhere (9). Briefly, the mice were anesthetized with isoflurane inhalation (4% for induction and 1.5%–2% for maintenance), but not ventilated. Eye ointment (Viscotears, Artelac) was applied during the procedure to protect the retina, and a small incision was made over the left chest to expose the fourth intercostal space. Following a small cut into the fourth intercostal space, the heart was gently mobilized out through the hole. The left anterior descending artery (LAD) was permanently ligated approximately 3 mm from its origin using a 6-0 silk suture. Successful ligation was indicated when the anterior wall of the LV turned pale. After ligation, the heart was immediately placed back into the chest cavity and the incisions closed. Immediately after surgery, the mice were treated with either P7 (8.5 mg/kg) or vehicle (H_2_O) through i.p. injections. Appropriate analgesic treatment (buprenorfin, 0.1 mg/kg) was administered, and the mice recovered under strict monitoring.

At 9 h post-LAD surgery, blood was collected through cardiac puncture for cytokine measurements. Then, at 3 days post-LAD surgery, blood was drawn from awake animals by a pinprick to the saphenous vein and collected into an EDTA-coated capillary tube (Sarstedt). At the end of the experiment (12 weeks post-LAD surgery), the mice were anesthetized with isoflurane inhalation (4% for induction and 1.5%–2% for maintenance). The left carotid artery was exposed and nicked, and the arterial blood was collected with 0.5 M EDTA (Fluka, Sigma-Aldrich). The EDTA blood was immediately placed on ice and centrifuged within 30 min at 2,000*g* (4°C) for 20 min to obtain platelet-poor plasma.

The collected hearts were either fixated in 4% formaldehyde and sliced into standardized 2-mm sections using a mouse heart slicer matrix (Zivic instruments, Pittsburg PA) for histology or separated into RV and LV. The LV was further separated into infarcted or non-infarcted tissue.

### Echocardiography

Echocardiography was performed under standardized conditions with the mice in a supine position, spontaneously breathing isoflurane (4% for induction and 1.5%–2% for maintenance) mixed with O_2_ via a mask. Eye ointment (Viscotears, Artelac) was applied during the procedure to protect the retina. Standard parasternal long axis images were acquired with the Vevo 3100 system and analyzed offline using Vevo LAB3.2 software (VisualSonics, Toronto, Ontario, Canada). For the evaluation of heart function, three consecutive M-mode cardiac cycles were analyzed and averaged. The various echocardiographic parameters were calculated as follows: fractional shortening (FS) = [left ventricular (LV) end-diastolic diameter (LVEDD) - LV end-systolic diameter (LVESD)/LVEDD)] × 100; ejection fracture (EF) = [stroke volume (SV)/end-diastolic volume (EDV)] × 100. For the evaluation of successful MI surgery, myocardial contractility was assessed using B-mode imaging along the long axis at a reduced speed. Both upper and lower walls were scrutinized, and uniformity across multiple mice was ensured. An autopsy examination of MI size was performed independently of the echocardiographic results. At the final analysis, these two observations were integrated to derive conclusive insights into infarct size. All echocardiographic measurements and analyses were performed blinded for treatment and group. For six individuals (*n* = 3 P7 and *n* = 3 H_2_O), at least one echocardiographic parameter could not be confidently measured, and they were excluded from the data set.

### Cytokine measurements

Plasma cytokine levels were determined by 23 cytokines (23-plex Assay, #M60009RDPD) BioPlex cytokine assays from Bio-Rad, in accordance with the instructions of the manufacturer, using the Bio-Plex Pro™ Reagent Kit III and Bio-Plex™ 200 System (Bio-Rad, Hercules, CA, USA).

### Histology, picrosirius red staining, and MI quantification

Heart sections were formalin-fixed, and a sample from a standardized location relative to the point of infarct was processed for histology. After dehydration and paraffin embedding, the sample was sliced into 5-µm-thick cross-sections, then deparaffinized in xylene, and rehydrated in EtOH (4× 100%, 2× 96%, 1× 80%, and 1× 50%). To stain for fibrosis, sections were incubated for 1 h in sirius red solution (Histolab Products AB, Gothenburg, Sweden), followed by two rinsing steps with acidified water. After two quick dips in 100% ethanol, the sections were rinsed in xylene and mounted with Eukitt (Sigma-Aldrich). The sections were scanned and evaluated in the z9 platform, an in-house-made software program for examining and quantitatively evaluating histological sections.

### Statistical analysis

GraphPad Prism version 9.0 (GraphPad Software, La Jolla, CA, USA) was used for statistical analysis. Differences between groups was assessed by unpaired, two-tailed Student’s *t*-test. Significance was considered at *p* < 0.05.

## Results

### Experimental set-up

**Figure 1:**
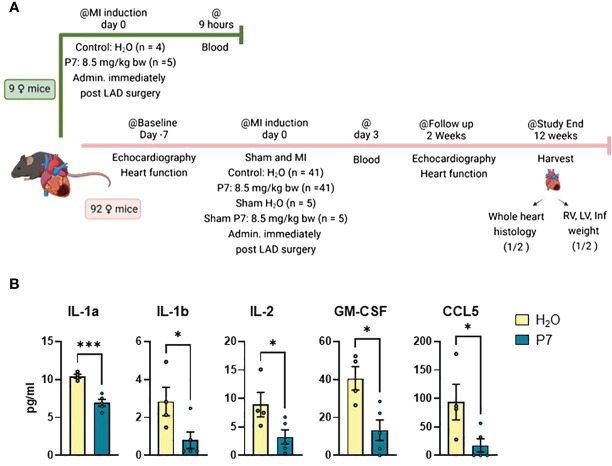
Early effects of P7 on myocardial inflammation post-myocardial infarction. **(A)** Schematic presentation of the experimental setup. A total of 101 female mice were entered in the study. Nine animals were allocated to examine the effect of P7 on initial inflammatory signaling after MI. P7 (*n* = 5) or H_2_O (control, *n* = 4) was injected intraperitoneally immediately after induction of myocardial infarction (MI) by permanent ligation of the left anterior descending artery (LAD). After 9 h, blood samples were collected by heart puncture. For the remaining 92 mice, echocardiography was performed 7 days pre-LAD surgery as baseline, before induction of permanent myocardial infarction (MI) (n = 41 P7, n = 41 H_2_O) or sham operation (n = 5 P7, n = 5 H_2_O). Blood samples were collected 3 days post-LAD surgery from the saphenous vein, and cardiac function was assessed by echocardiography 2 weeks post-LAD surgery. At the end of the study, blood samples were collected from the left carotid artery. For half of the permanently LAD-ligated animals, heart was collected for histology, while the latter hearts were dissected into the right ventricle (RV), left ventricle (LV), and infarcted tissue. **(B)** Significantly regulated cytokines and chemokines measured by multiplex (*n* = 4 to 5) 9 h post-LAD surgery. Data are presented as mean ± SEM. *p < 0.05, ***p < 0.005, unpaired Student’s *t*-test. The full overview of cytokine measured is given in **Supplementary Figure 1**.

**Figure 1A** illustrates the experimental setup. In total, 101 female C57BL/6J mice (age 8 weeks) were used in the study. Nine animals were allocated to examine the effect of P7 on the initial inflammatory signaling after MI. Briefly, acute experimental MI was induced by manually exposing the heart without intubation through a small incision and permanent occlusion of the left anterior descending artery (LAD) by ligation. Immediate discoloration of LV indicated an occlusion (9). The test groups received either i.p. P7 (8.5 mg/kg body weight) or a similar volume of H_2_O immediately after surgery (P7: *n* = 5, H_2_O: *n* = 4). At 9 h post-LAD surgery, the mice were sacrificed through cardiac puncture for blood collection to determine the initial effect of P7 administration on the inflammatory pathways.

For the remaining 92 mice, a baseline echocardiography was performed before LAD surgery as described above. From this group, eight mice died during surgery and 16 mice reached human end-points within 24 h after LAD surgery (13 H_2_O and 11 P7). For the surviving mice, successful MI was confirmed by echocardiography 1 day after LAD surgery and at the end of the experimental period (12 weeks post-LAD surgery). Based on these assessments, 12 mice were excluded due to failure of inducing MI (seven H_2_O and five P7). At day 2 post-LAD surgery, one mouse reached human end-points (1 P7), and at day 4 post-LAD surgery, two mice from the MI H_2_O group died from cardiac rupture. Finally, the included groups consisted of sham H_2_O (*n* = 5) and sham P7 (*n* = 5) as well as MI H_2_O (*n* = 21) and MI P7 (*n* = 22). The sham operated mice went through the same procedure, except ligating the LAD.

### P7 inhibits initial inflammatory signaling after MI

At 9 h post-LAD surgery, blood was collected through cardiac puncture for cytokine measurements. Treatment with P7 significantly dampened the levels of the proto-typical inflammatory cytokines/chemokine IL-1α, IL-1β, and CCL5, T cell growth factor IL-2, and granulocyte-macrophage colony-stimulating factor (GM-CSF) (**Figure 1B**). An analysis was conducted using the multiplex cytokine assays, and an overview over all measured cytokines is shown in **Supplementary Figure 1**.

In contrast to the short-term effects of P7 on inflammation in cardiac blood, no effects of P7 were seen in plasma at 3 days post-LAD surgery (**Supplementary Figure 2**). Although the blood samples were collected from the saphenous vein and not directly from the heart as in the analyses 9 h post-LAD surgery, the lack of cytokine regulation at this time point indicates that inhibition of inflammation caused by P7 only occurs at an early time point post-LAD surgery, probably due to the short half-life of P7. Our data suggest that P7 dampens the initial and damaging wave of inflammation following LAD surgery-induced MI, without compromising inflammation during the healing and remodeling process.

### P7 reduces infarct size and improves cardiac function after MI

**Figure 2:**
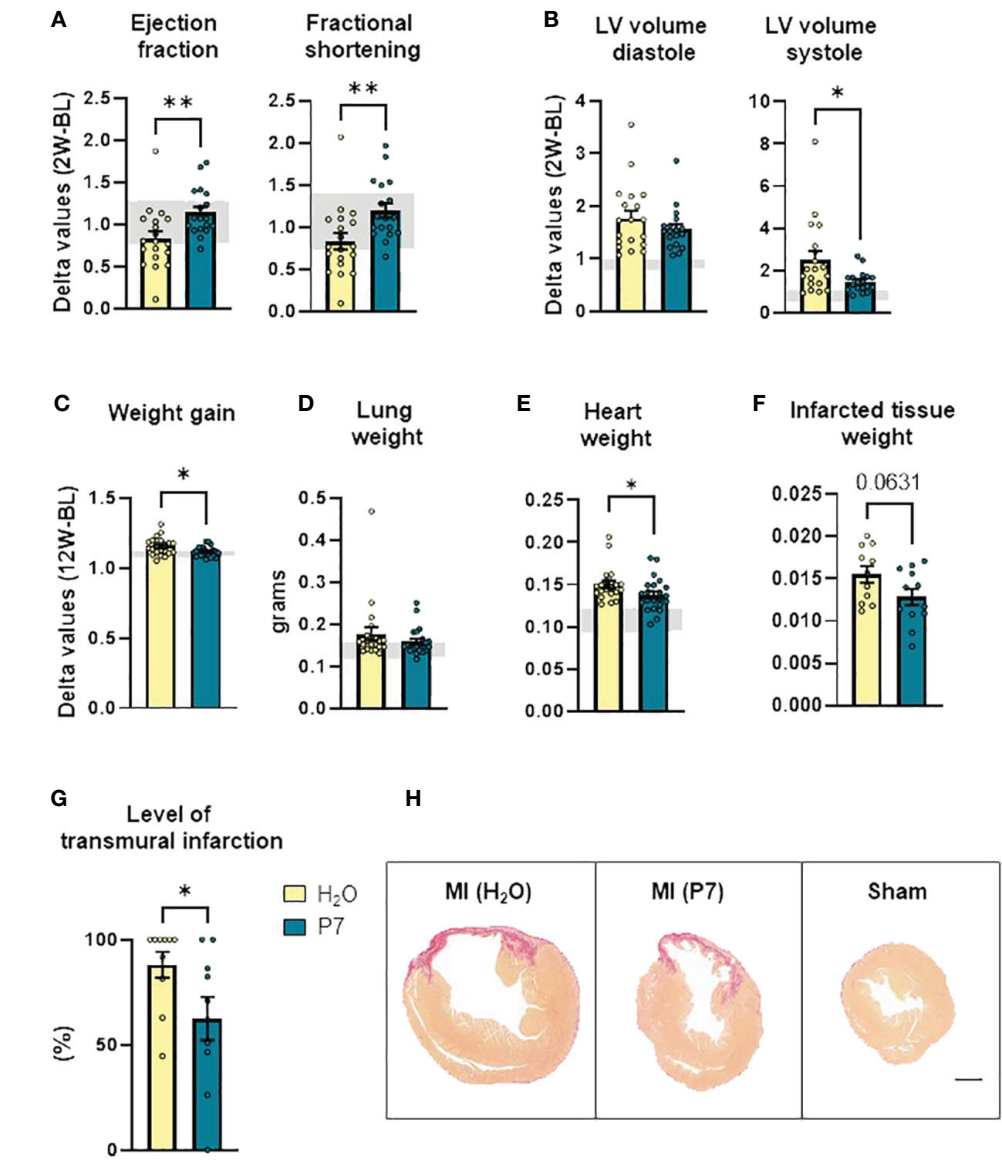
Late effects of P7 on myocardial remodeling post-myocardial infarction. **(A)** Ejection fraction and fractional shortening (*n* = 18 to 19). **(B)** Left ventricle volume in diastole and systole, calculated based on echocardiographic measurements 2 weeks post-left anterior descending artery surgery (*n* = 18 to 19). **(C)** Total weight gain during the experimental period (*n* = 21 to 22). **(D)** Lung weight (*n* = 21 to 22), **(E)** heart weight (*n* = 21 to 22), and **(F)** infarcted tissue weight (*n* = 11 to 12). **(G)** Level of transmural infarcted tissue calculated in picrosirius red-stained heart sections (*n* = 10). **(H)** Representative images of picrosirius red-stained heart sections from MI-P7, MI-H_2_O, and Sham groups, respectively (scale bar: 1,000 µm). Data are shown as mean ± SEM. *p* < 0.1 is written in the figure, *p < 0.05, **p < 0.01, unpaired Student *t*-test. The gray areas represent upper and lower values within the sham H_2_O-group.

We measured cardiac function by echocardiography at baseline (BL) and two weeks after LAD surgery. As shown in **Figure 2A**, mice receiving P7 had significantly less deteriorated cardiac function as measured by LV ejection fraction (LVEF) and fractional shortening compared to controls receiving H_2_O. In line with this, the P7-treated group show less LV dilation during systole (**Figure 2B**). All echocardiographic measurements for both BL and after 2 weeks are listed in **Supplementary Table 1**.

### P7 attenuates body weight gain and reduces the proportion of transmural MI

At the end of the study, i.e., 12 weeks post-LAD surgery, the mice treated with P7 gained less body weight compared to the control group (H_2_O) (**Figure 2C**), potentially reflecting less fluid retention in these mice. In contrast, lung weight for most mice was within the range of sham operated animals, suggesting no overt heart failure (**Figure 2D**). The P7-treated animals had a significant reduction in heart weight compared to H_2_O controls, suggesting attenuated myocardial hypertrophy (**Figure 2E**). For half of the animals, infarcted tissue was separated and weighted, showing a tendency toward decreased infarcted tissue weight in the P7-treated group (*p* = 0.06) (**Figure 2F**). For the other half of the animals, the hearts were fixed in formalin, paraffin-embedded, and cross-sectioned. The infarcted area was analyzed using picrosirius red-stained sections, and the level of transmural infarction was calculated in a section 2 mm caudal from the ligation. In line with less deterioration of heart function and decreased infarct size weight in mice treated with P7, a significantly lower proportion of these animals had LV transmural infarcts, involving all three layers of the myocardium (**Figures 2G, H**), further suggesting less myocardial damage in the P7-treated mice.

## Discussion

In the present study, we demonstrated that P7, a novel SLAMF1-derived peptide, dampens pro-inflammatory signaling, likely through an inhibitory effect on TLR4-mediated signaling, and has protective properties against experimentally induced MI. Mice receiving P7 immediately after LAD ligation had less deteriorated LV function, less LV systolic dilation, attenuated weight gain, and a reduced proportion of LV transmural infarct compared to control mice. The effect of P7 on inflammation, as assessed by the levels of inflammatory cytokines in blood, was evident within hours post-LAD surgery, but not after 3 days, reflecting the short half-life of P7. These properties could present a significant advantage in targeted therapy for inflammation after MI. They would ensure the blockage of the initial harmful inflammatory wave while preserving the subsequent phase of inflammation, which is considered necessary for proper infarct healing and adaptive remodeling (10).

Anti-inflammatory therapy can focus on mitigating the impact of inflammatory cytokines by using antibodies targeting these molecules or by inhibiting their receptors. Another appealing approach could involve inhibiting upstream signaling pathways to reduce the production and release of inflammatory mediators. Several studies have demonstrated an emerging role for TLRs as critical modulators in both cell survival and tissue injury in the heart (11, 12). Of importance to note is that TLR4, which is more highly expressed compared to other TLRs in the heart, plays a crucial role in myocardial inflammation. This has been demonstrated in several studies, including preclinical investigations involving various knock-out models where the deletion of TLR4 had a protective effect against cardiac injury (13). However, a permanent or long-term inhibition of the TLR4 pathways will compromise not only the initial inflammatory responses but also proper and beneficial immune responses during the healing process (14). In a recent study in a rat model of cardiac I/R, a specific aptamer antagonist blocked TLR4 signaling after reperfusion and showed protective effects against cardiac injury 7 days post-MI (15), supporting the beneficial effects of targeting this pathway early after MI as shown in the present study by P7.

We have recently identified the interaction domains of SLAMF1 and TRAM and shown that the SLAMF1–TRAM protein–protein interaction is essential for the regulation of TLR4–TRIF–TRAM-dependent signaling in human innate immune cells (16). We have also shown that the SLAMF1-derived peptide P7 blocks the interaction between SLAMF1 and TRAM proteins (6). Moreover, P7 peptide also blocks TIRAP/Mal and MyD88 interaction, while it does not impact the interaction between TLR4 and TIRAP, and neither does it affect TLR2 or STING signaling. The immunological consequences of P7 inhibition of these and potentially other unrevealed protein–protein interactions are currently unclear. However, one could hypothesize that P7-mediated inhibition could result in a more targeted anti-inflammatory effect, avoiding blocking of potential anti-inflammatory pathways that may promote healing (17).

The present study has certain limitations, notably the absence of tibia measurements for normalization of metric data, the absence of data regarding myocardial inflammation, and the inclusion of only female subjects. The inclusion of only female mice was based on the knowledge that male mice are more at risk of cardiac rupture after LAD surgery (18, 19). To explore the beneficial effects of P7 on preserving cardiac function after LAD surgery, the choice of female mice complied to the best practice of refinement, reduction, and replacement (3Rs), and potential gender differences will be addressed in a future study. Moreover, in order to more precisely mimic the situation in the clinical setting in myocardial infarction patients, future studies should also examine the effect of P7 on ischemia/reperfusion. Despite these limitations, our findings demonstrate a promising effect of the SLAMF1-derived peptide P7 in a pre-clinical MI model. The results suggest that upstream inhibition of TLR signaling, with subsequent reduction in cytokine production and release, led to a short-term anti-inflammatory effect. Since the anti-inflammatory effect is short-lived, it may not reduce the beneficial effects of inflammation on infarct healing and adaptive remodeling, ultimately resulting in improved cardiac function. Finally, the aim of the present study was to clarify if the immediate treatment with P7 after LAD surgery could preserve more cardiac function after the healing and remodeling phase. Biopsies acquired this long (12 weeks) after the diminished effect of P7 was unfortunately not suitable to address the protective mechanism depending on P7 administration. This will be clarified in a future study.

## Data availability statement

The raw data supporting the conclusions of this article will be made available by the authors, without undue reservation.

## Ethics statement

The animal study was approved by The Norwegian Food Safety Authority. The study was conducted in accordance with the local legislation and institutional requirements.

## Author contributions

MO: Writing – review & editing, Writing – original draft, Visualization, Investigation, Formal Analysis, Conceptualization. XK: Writing – review & editing, Writing – original draft, Visualization, Investigation, Formal Analysis, Conceptualization. ML: Writing – review & editing, Visualization, Investigation, Formal Analysis, Conceptualization. KHL: Writing – review & editing, Visualization, Validation, Formal Analysis. YS: Writing – review & editing, Investigation. OK: Writing – review & editing, Investigation. HA: Writing – review & editing. JØ: Writing – review & editing, Visualization, Formal Analysis. SH: Writing – review & editing, Investigation. PA: Writing – review & editing, Writing – original draft, Supervision, Conceptualization. LR: Writing – review & editing, Investigation, Formal Analysis. TE: Writing – review & editing, Supervision, Funding acquisition, Conceptualization. MY: Writing – review & editing, Investigation, Funding acquisition, Formal Analysis, Conceptualization. BH: Writing – review & editing, Supervision, Resources, Project administration, Funding acquisition, Conceptualization.

## Supplementary material

Supplementary Figure 1 Cytokine regulation 9 h post-myocardial infarction, extended. Cytokine regulation 9 h post-left anterior descending artery surgery in mice receiving P7 (*n* = 5) or H_2_O (*n* = 4). This figure is an extension of data presented in **Figure 1B**.

Supplementary Figure 2 Cytokine regulation 3 days post-myocardial infarction. Cytokine regulation 3 days post- left anterior descending artery surgery in mice receiving P7 (*n* = 22) or H_2_O (*n* = 21). The gray areas represent upper and lower values within the sham H_2_O-group. KC, keratinocyte chemoattractant. IL-1b, IL-2, IL-3, IL-4, IL-9, INF-g, MIP-1b, MCP-1, and GM-CSF were also measured but were undetectable in most samples.
