# Supplementary figures and images for: SLAMF1-derived peptide exhibits cardio protection after permanent left anterior descending artery ligation in mice
Source: Front Immunol. 2024 Apr 15;15:1383505. doi: 10.3389/fimmu.2024.1383505 (PMC11056545; doi:10.3389/fimmu.2024.1383505)

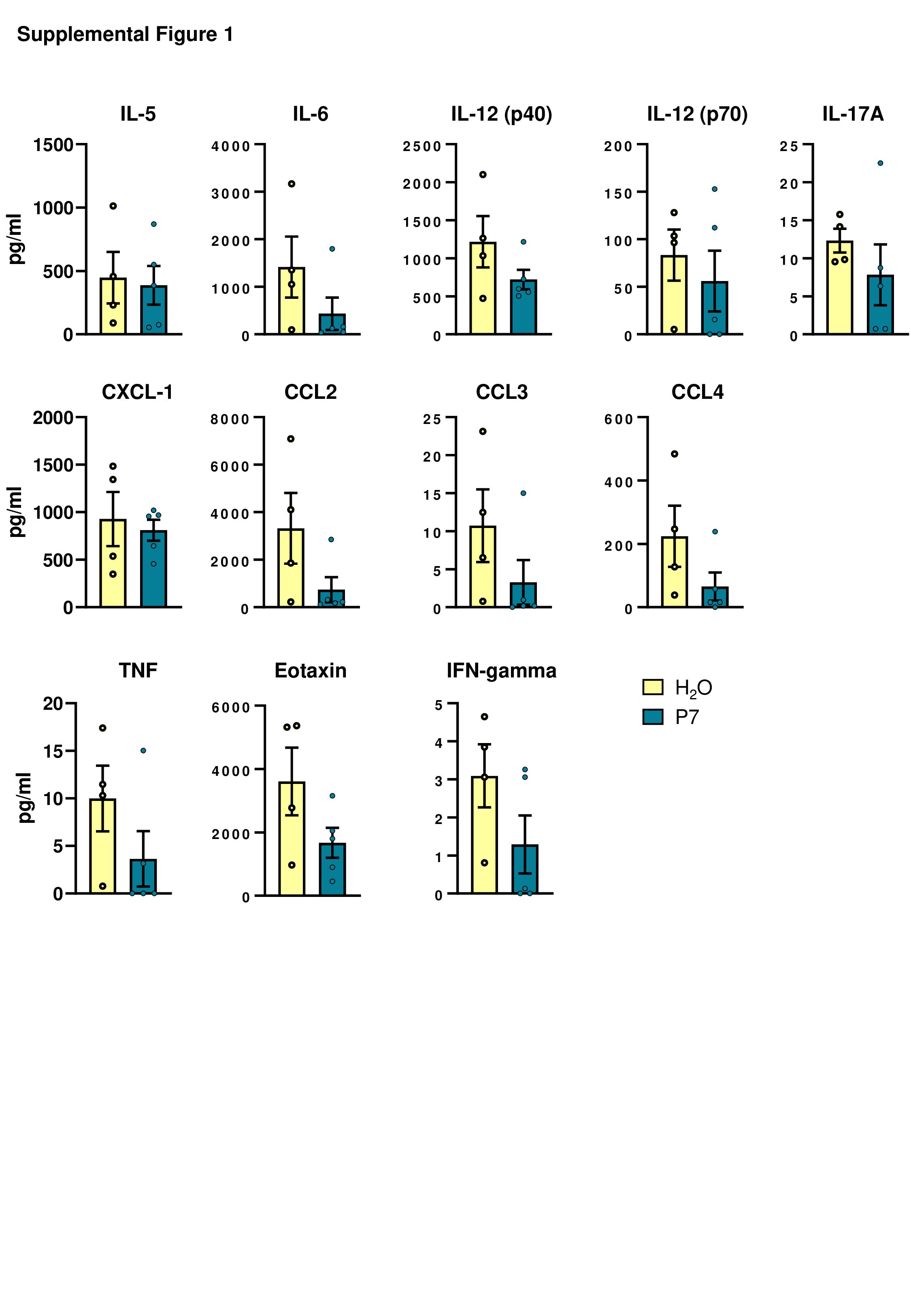

Supplement: Supplementary Figure 1 — Cytokine regulation 9 h post-myocardial infarction, extended. Cytokine regulation 9 h post-left anterior descending artery surgery in mice receiving P7 (n = 5) or H2O (n = 4). This figure is an extension of data presented in Figure 1B . [file Image_1.jpeg]

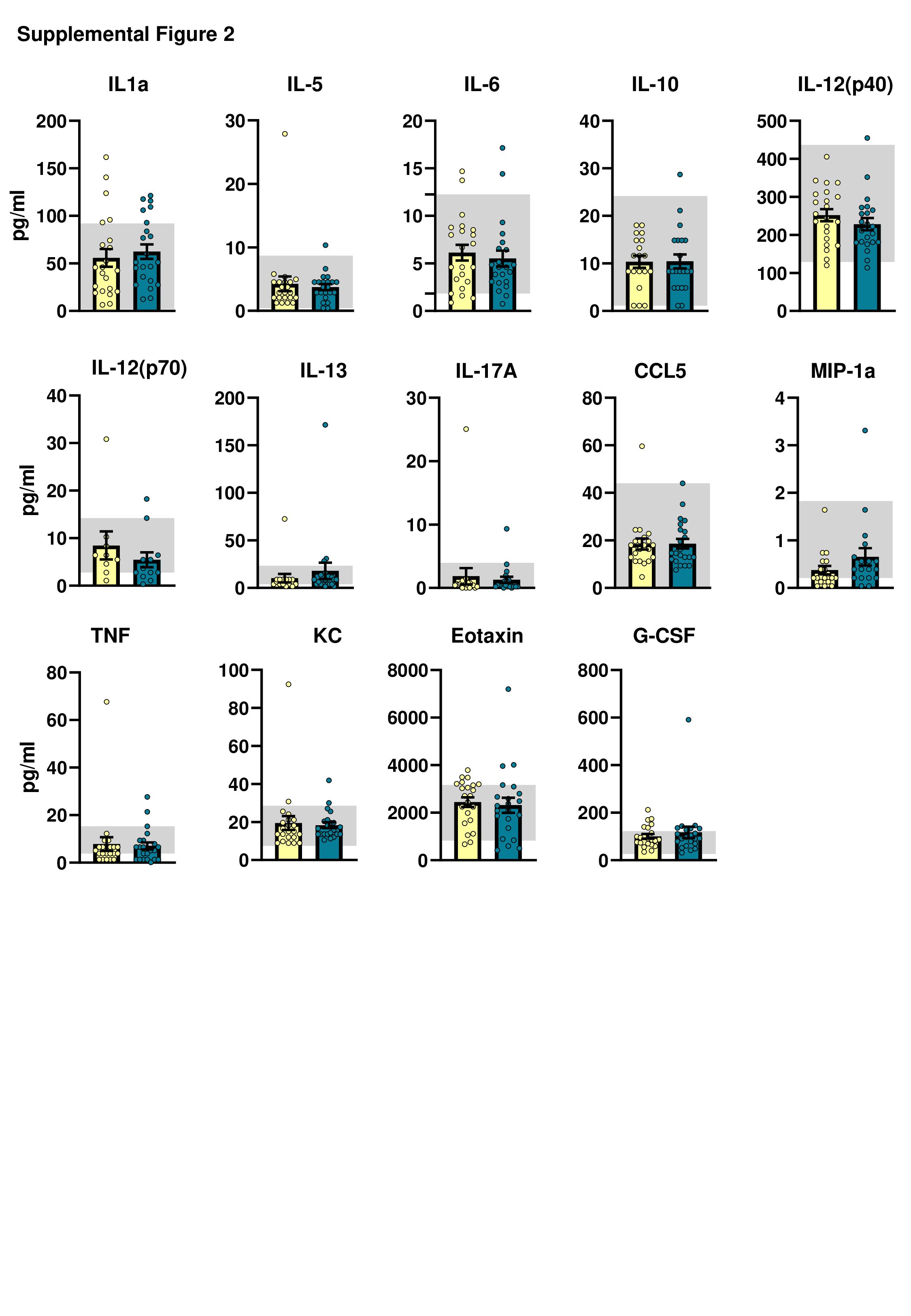

Supplement: Supplementary Figure 2 — Cytokine regulation 3 days post-myocardial infarction. Cytokine regulation 3 days post- left anterior descending artery surgery in mice receiving P7 (n = 22) or H2O (n = 21). The gray areas represent upper and lower values within the sham H2O-group. KC, keratinocyte chemoattractant. IL-1b, IL-2, IL-3, IL-4, IL-9, INF-g, MIP-1b, MCP-1, and GM-CSF were also measured but were undetectable in most samples. [file Image_2.jpeg]
