# Supplementary material for: SLAMF1-derived peptide exhibits cardio protection after permanent left anterior descending artery ligation in mice
Source: Front Immunol. 2024 Apr 15;15:1383505. doi: 10.3389/fimmu.2024.1383505 (PMC11056545; doi:10.3389/fimmu.2024.1383505)
Supplement: Supplementary file 3 [file Table_1.docx]

| **Table S1. Animal characteristics and echocardiographic measurements** | | | | | | | | | | |  |  |  |  |  |
| --- | --- | --- | --- | --- | --- | --- | --- | --- | --- | --- | --- | --- | --- | --- | --- |
|  |  |  |  |  |  |  |  |  |  |  |  |  |  |  |  |
|  | **Sham** | | | | | |  | **MI** | | | | | | |  |
|  |  | **H_2_0** |  |  | **P7** |  |  |  | **H_2_0** |  |  |  | **P7** |  | p-value |
|  | Mean |  | SE | Mean |  | SE |  | Mean |  | SE |  | Mean |  | SE |  |
| **Characteristics** |  |  |  |  |  |  |  |  |  |  |  |  |  |  |  |
| n |  | 5 |  |  | 5 |  |  |  | 21 |  |  |  | 22 |  |  |
| BW,g BL | 21.48 | ± | 2.23 | 21.42 | ± | 1,27 |  | 21.76 | ± | 1.19 |  | 21.69 | ± | 1.40 |  |
| BW, g End | 23.66 | ± | 2.43 | 23.74 | ± | 1.81 |  | 25.04 | ± | 1.75 |  | 24.20 | ± | 1.71 |  |
| HW, mg | 103.5 | ± | 12.55 | 103.5 | ± | 10.82 |  | 149.9 | ± | 19.7 |  | 137.9 | ± | 19.9 | 0.053 |
| HW/BW BL | 4.81 | ± | 0.20 | 4.53 | ± | 0.41 |  | 6.92 | ± | 1.04 |  | 6.37 | ± | 0.90 | 0.069 |
| LW | 141.4 | ± | 16.99 | 150.4 | ± | 17.56 |  | 177.0 | ± | 72.4 |  | 159.2 | ± | 31.9 |  |
| LW/BW BL | 6.57 | ± | 0.27 | 7.00 | ± | 0.61 |  | 8.16 | ± | 3.26 |  | 7.33 | ± | 1.39 |  |
| SW | 93.9 | ± | 11.26 | 96.0 | ± | 14.61 |  | 98.1 | ± | 8.9 |  | 91.9 | ± | 10.7 | * |
| SW/BW BL | 4.34 | ± | 0.16 | 0.50 | ± |  |  | 4.53 | ± | 0.48 |  | 4.27 | ± | 0.44 | 0.076 |
|  |  |  |  |  |  |  |  |  |  |  |  |  |  |  |  |
| n |  | 3 |  |  | 3 |  |  |  | 11 |  |  |  | 12 |  |  |
| RVW | 17.6 | ± | 3.19 | 19.8 | ± | 20.8 |  | 25.2 | ± | 3.9 |  | 24.0 | ± | 6.2 |  |
| RVW/BW BL | 0.81 | ± | 0.14 | 0.89 | ± | 0.18 |  | na |  |  |  | na |  |  |  |
| LVW | 86.0 | ± | 11.9 | 90.1 | ± | 6.8 |  | 117.4 | ± | 7.1 |  | 110.5 | ± | 14.1 |  |
| LVW/BW BL | 3.95 | ± | 0.22 | 4.06 | ± | 0.43 |  | 5.41 | ± | 0.41 |  | 51.21 | ± | 0.60 |  |
| IW | na |  |  | na |  |  |  | 15.51 | ± | 3.26 |  | 12.84 | ± | 3.27 |  |
| IW/BW BL | na |  |  | na |  |  |  | 0.72 | ± | 0.18 |  | 0.60 | ± | 0.17 |  |
|  |  |  |  |  |  |  |  |  |  |  |  |  |  |  |  |
| **M-Mode** |  |  |  |  |  |  |  |  |  |  |  |  |  |  |  |
| **Baseline** |  |  |  |  |  |  |  |  |  |  |  |  |  |  |  |
| n |  | 5 |  |  | 5 |  |  |  | 23 |  |  |  | 21 |  |  |
| IVSd, mm | 0.873 | ± | 0.09 | 0.758 | ± | 0.15 |  | 0.830 | ± | 0.13 |  | 0.756 | ± | 0.09 | * |
| IVSs | 1.149 | ± | 0.12 | 1.041 | ± | 0.20 |  | 1.074 | ± | 0.12 |  | 1.022 | ± | 0.14 |  |
| LVDd | 3.660 | ± | 0.18 | 3.898 | ± | 0.13 | * | 3.729 | ± | 0.21 |  | 3.699 | ± | 0.25 |  |
| LVDs | 2.713 | ± | 0.22 | 2.943 | ± | 0.17 |  | 2.738 | ± | 0.36 |  | 2.797 | ± | 0.31 |  |
| PWd | 0.939 | ± | 0.13 | 0.717 | ± | 0.12 | * | 0.819 | ± | 0.14 |  | 0.812 | ± | 0.17 |  |
| PWs | 1.157 | ± | 0.14 | 0.936 | ± | 0.11 | * | 1.090 | ± | 0.16 |  | 1.037 | ± | 0.20 |  |
| LV Vol;d | 56.81 | ± | 6.88 | 65.93 | ± | 5.17 | * | 59.48 | ± | 7.97 |  | 58.48 | ± | 9.02 |  |
| LV Vol;s | 27.58 | ± | 5.80 | 33.56 | ± | 4.63 |  | 28.73 | ± | 9.53 |  | 30.03 | ± | 8.23 |  |
| VEF, % | 51.8 | ± | 4.4 | 48.8 | ± | 8.4 |  | 52.6 | ± | 10.5 |  | 48.9 | ± | 9.3 |  |
| LVFS, % | 25.9 | ± | 2.6 | 24.4 | ± | 5.4 |  | 26.8 | ± | 6.3 |  | 24.4 | ± | 5.7 |  |
|  |  |  |  |  |  |  |  |  |  |  |  |  |  |  |  |
|  |  |  |  |  |  |  |  |  |  |  |  |  |  |  |  |
| **Follow up** |  |  |  |  |  |  |  |  |  |  |  |  |  |  |  |
| n |  | 5 |  |  | 5 |  |  |  | 21 |  |  |  | 19 |  |  |
| IVSd, mm | 0.829 | ± | 0.07 | 0.898 | ± | 0.22 |  | 0.861 | ± | 0.15 |  | 0.908 | ± | 0.14 |  |
| IVSs | 1.152 | ± | 0.12 | 1.154 | ± | 0.17 |  | 1.257 | ± | 0.23 |  | 1.378 | ± | 0.22 |  |
| LVDd | 3.490 | ± | 0.22 | 3.570 | ± | 0.50 |  | 4.664 | ± | 0.52 |  | 4.527 | ± | 0.287 | 0.079 |
| LVDs | 2.545 | ± | 0.42 | 2.43 | ± | 0.26 |  | 3.718 | ± | 0.65 |  | 3.212 | ± | 0.32 | ** |
| PWd | 0.801 | ± | 0.15 | 0.802 | ± | 0.14 |  | 0.876 | ± | 0.17 |  | 0.888 | ± | 0.15 |  |
| PWs | 1.100 | ± | 0.14 | 1.100 | ± | 0.11 |  | 1.050 | ± | 0.22 |  | 1.123 | ± | 0.24 |  |
| LV Vol;d | 50.76 | ± | 7.68 | 54.65 | ± | 17.31 |  | 102.25 | ± | 26.73 |  | 89.02 | ± | 13.52 | 0.065 |
| LV Vol;s | 24.20 | ± | 10.30 | 21.15 | ± | 5.95 |  | 61.49 | ± | 28.63 |  | 41.93 | ± | 9.67 | ** |
| LVEF, % | 53.21 | ± | 13.91 | 59.78 | ± | 9.12 |  | 41.6 | ± | 12.1 |  | 53.2 | ± | 6.4 | *** |
| LVFS, % | 27.27 | ± | 8.78 | 31.42 | ± | 6.65 |  | 20.6 | ± | 6.6 |  | 27.4 | ± | 4.1 | *** |
|  |  |  |  |  |  |  |  |  |  |  |  |  |  |  |  |
|  |  |  |  |  |  |  |  |  |  |  |  |  |  |  |  |
